# The Use of the Probiotic *Lactiplantibacillus plantarum* 299v in the Technology of Non-Dairy Ice Cream Based on Avocado

**DOI:** 10.3390/foods10102492

**Published:** 2021-10-18

**Authors:** Ada Krawęcka, Justyna Libera, Agnieszka Latoch

**Affiliations:** 1 Department of Plant Food Technology and Gastronomy, University of Life Sciences in Lublin, 20-704 Lublin, Poland; ada.krawecka@gmail.com; 2 Department of Animal Food Technology, University of Life Sciences in Lublin, 20-704 Lublin, Poland; agnieszka.latoch@up.lublin.pl

**Keywords:** functional food, non-dairy ice cream dessert, probiotic, *Lactiplantbacillus plantarum* 299v

## Abstract

Food enriched with probiotics and prebiotics belong to the class of novel foods. Functional food, apart from its nutritional function, has an additional pro-health effect. The aim of the presented study was to create a concept of a functional dessert—avocado-based non-diary ice cream enriched with probiotic bacteria *Lactiplantibacillus plantarum* 299v. The product was tested for the survival of bacteria in various conditions, and the influence of the probiotic on the physicochemical and organoleptic properties of non-dairy ice cream was assessed. The dessert with probiotic throughout the storage period (8 weeks) kept the therapeutic minimum defined for probiotic food products. It was found that the addition of the probiotic did not deteriorate either the color or the sensory profile of the dessert. There was also no increase in the redox potential nor the acidity of the product with the addition of a probiotic.

## 1. Introduction

Today, access to highly processed food is common, therefore carrying the risk of disturbances in carbohydrate and fat metabolism, and, ultimately, also leads to the development of the obesity epidemic. These are far-reaching effects of the growing wealth of the population, especially the population of highly developed countries. It is a Western-style diet based on flour and sugar, rich in monosaccharides, glucose-fructose syrup, and saturated fats [1]. Negative eating patterns can disrupt the microbiome, and this is associated with chronic inflammation [2]. Dysbiosis may be a pathogenetic factor in the development of type 2 diabetes, dyslipidemia, chronic kidney diseases, and arterial hypertension [3]; therefore, it is necessary to pay more attention to the important role played by the gastrointestinal microbiota. 

The proper functioning of the intestines, and, more specifically, the colonizing microorganisms, can be supported by probiotic therapy. A good solution is including in daily diet products that are source of probiotic lactic acid bacteria (LAB) with prebiotics that feed them. The health benefits of consuming probiotics and prebiotics are well known and include obesity and the metabolic syndrome prevention [4]. Products of this class are very popular among consumers. However, it should be remembered that the functional properties of probiotics depend on the specific strain and may vary considerably, even within the same genera. The most widely used and well-studied species are *Lactobacillus* and *Bifidobacterium* [5]. *Lactiplantibacillus plantarum* is commonly found in vegetables and fermented products. It is also present in the mucosa of the human small intestine, and its functional properties have been confirmed by in vitro and in vivo tests [6,7,8,9]. The probiotic strain *L. plantarum* 299v is used as a starter culture in the technology of fermented food of plant origin [10]. Although the application of probiotics in dairy products has been widely explored in the literature, ice cream is a relatively innovative matrix for the application of probiotics; thus, a review about its potential as probiotic food carrier could be very helpful [11]. Ice cream is a product with great potential to act as a food carrier for bacteria, due to the presence of milk sugar, which is an excellent breeding ground for bacteria [12]. The development of the technology of non-dairy ice cream containing probiotic bacteria, however, requires overcoming certain technological difficulties related to various stages of their processing [11].

There are milk-based ice cream and frozen dairy-free desserts made from soy or coconut vegetable proteins and traditional fruit-based sorbets [13,14]. Avocado paste was also used as a raw material, as a fat substitute in the technology of frozen desserts [15]. As there are few scientific reports on the use of probiotics LAB in ice-cream dessert technology, this research was undertaken. The aim of the work was to create a concept of a functional dessert—avocado-based non-dairy ice cream enriched with probiotic *L. plantarum* 299v.

## 2. Materials and Methods

### 2.1. Raw Materials for an Non-Dairy Ice Cream Dessert

The research material was an avocado-based non-dairy ice cream dessert with the addition of banana and lime juice. The basic ingredients of the dessert were fresh fruit—banana and avocado (in a 2:1 ratio), obtained from a local grocery store. The choice of plant raw materials was guided by the fact that the banana is a sweet fruit (contains on average about 22 g of digestible carbohydrates in 100 g of product [16]) and, thus, could give the product a sweet taste without the need to add sweetener or sugar. The polysaccharides in bananas are likely to help maintain LAB viability during freezing [17]. Avocados, on the other hand, can provide an optimal, creamy consistency of dessert by the presence of unsaturated fats (approximately 13 g/100 g) [16]. The presence of fat may be used as a cryoprotectant [17]. As both raw materials are prone to color changes during processing, lime juice was added to stabilize the color. 

### 2.2. Preparation of the Probiotic Inoculum

A probiotic inoculum was added to the product in the form of a lyophilized or reconstituted forms strain of *Lactiplantibacillus plantarum* 299v (DSM 9843) (pharmaceutical preparation in capsules, Sanprobi IBS, Sanum, Szczecin, Poland). According to the manufacturer’s declaration, one capsule of Sanprobi IBS contains at least 10 billion colony forming units (CFU). The preparation of the probiotic began 24 h before its application to the non-dairy ice cream dessert. All laboratory equipment used for this process was autoclaved at 121 °C (for 20 min). Exactly 90 g of the peeled banana were blended (Hand blender BEKO, Istanbul, Turkey) and put into 3 jars which were then autoclaved (121 °C for 20 min). In this way, a matrix for the probiotic was obtained. Next, the jars were cooled to 30 °C. One capsule of Sanprobi IBS was dissolved in 1 mL of sterile phosphate buffered saline (PBS), pH 7.4, and was added to the first jar (Lb1) only. Pure lyophilizate (content of 1 capsule without PBS) was added to the second jar (Lb2). The control sample (CON) was peeled banana with addition of 1 mL of sterile PBS solution.

### 2.3. Preparation of the Non-Dairy Ice Cream Desserts

**Figure 1:**
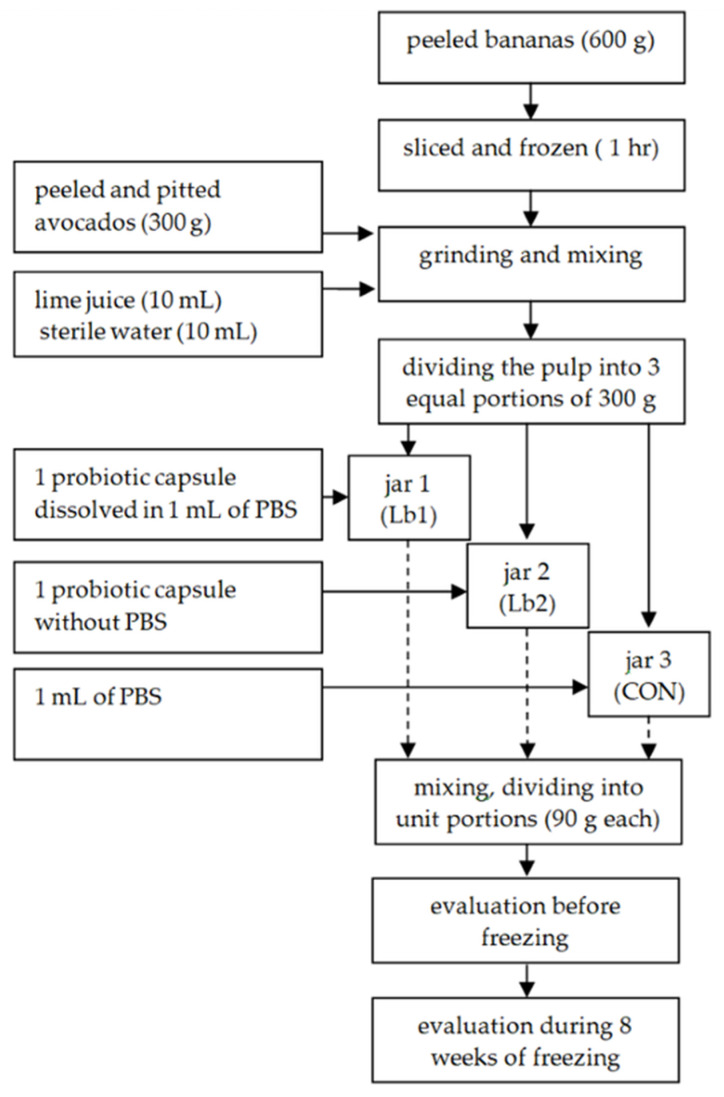
Technological scheme of non-dairy ice cream desserts.

The technological scheme of the product is shown in Figure 1. To produce non-dairy ice cream desserts (weight after peeling), the following were used: avocados (300 g), bananas (600 g), lime juice (10 mL), and 10 mL of sterile water. The bananas were peeled, sliced 1 cm thick, placed in a polyethylene bag, and frozen for 1 h. After this time, the avocados were peeled and pitted. Avocados, frozen bananas, lime juice, and water were placed in a blender and then minced for 3 min until a smooth, lump-free consistency was obtained. The fruit pulp was divided into 3 parts, each weighing about 300 g. The probiotic inoculum in PBS solution was added to the first (Lb1), the probiotic lyophilizate was added to the second (Lb2), and the third part (CON) contained pulp only. After mixing, the dessert samples were divided into unit portions weighing 90 g each. For this purpose, a weighed portion was transferred to sterile plastic containers and frozen at −23 °C. One batch was tested immediately after production (before freezing). The remaining lots were tested twice during an 8-week freezing period.

### 2.4. Estimating the Energy and Nutritional Value of an Non-Dairy Ice Cream Dessert

To assess the energy and nutritional value of an non-dairy ice cream dessert, the tables of nutritional values for banana and avocado [16] and the composition of our own recipe were used. The calculations were performed on a spreadsheet, where the individual nutritional values (protein, carbohydrate, and fat content) for each ingredient were entered, and the values were summed and divided by portion. It was assumed that 100 g of banana provide 97 kcal (412 kJ), and 100 g of avocado—169 kcal (696 kJ) [16]. The nutritional value of both fruits is presented in Table 1. 

### 2.5. Physico-Chemical Parameters

The pH value of the non-dairy ice cream dessert was measured using a digital pH meter (CPC-501; Elmetron, Zabrze, Poland) equipped with a combined pH glass electrode (ERH-111; Hydromet, Gliwice, Poland) in a suspension (sample/distilled water 1:10) homogenized for 1 min using a disperser (T25 Basic ULTRA-TURRAX; IKA, Staufen, Germany). The oxidation-reduction potential (Eh) was measured in a homogenate containing 10 g of sample and 30 mL of deionized water. Eh measurements of the homogenates were taken using digital pH-meter (CPC-501; Elmetron, Zabrze, Poland) set to the millivolt scale and equipped with platinum redox electrode (ERPt-13; Hydromet, Gliwice, Poland). All measurements were performed in triplicate and expressed as a mean ± standard deviation (SD).

### 2.6. Microbiological Analysis

The total viable counts (TVC) were quantified by means of the colony-count technique (PN-EN ISO 4833-1:2013-12) [18]. The number of LAB was determined according to PN-ISO 15214:2002 [19]. The counts were expressed as the log of colony-forming units (CFU) per gram of sample. Microbiological analyses were carried out at the Agrolab Group Laboratory (Dęblin, Poland). All measurements were performed in triplicate and expressed as a mean ± standard deviation (SD).

### 2.7. Color Evaluation of Non-Dairy Ice Cream Dessert 

The color of a dessert was examined using the instrumental method—reflectance (X-Rite Color 8200 spectrophotometer, X-Rite Inc., Grand Rapids, MI, USA). The conditions were 13 mm port size, illuminant D65 and 10° standard observer. The X-Rite’s white and black standards were used to calibrate the spectrophotometer. To determine the color parameters of the non-dairy ice cream dessert, a portion (5 g) was placed in a clean glass beaker, and then the beaker was placed in the measuring gap. The results were expressed on the CIEL*a* b* scale, where L* means lightness, and a* means green-red chromaticity, while b* means blue-yellow. All measurements were performed fifteen times and expressed as a mean ± standard deviation (SD).

The total color difference (ΔE*) was calculated based on ΔL*, Δa*, and Δb* results for each sample depending on the storage time in the freezer, as follows: ΔE* = ((ΔL*)^2^ + (Δa*)^2^ + (Δb*)^2^)^1/2^. The total color difference was interpreted as follows: invisible (0 < ∆E < 1), very small (1 < ∆E < 3.5), clear (3.5 < ∆E < 5), and large (∆E > 5).

### 2.8. Sensory Evaluation

The non-dairy ice cream samples were stored at −23 °C for 8 weeks, and sensory assessments were performed by 30 panelists (students at University of Life Sciences in Lublin, Poland). All samples were coded with two random digit numbers, and the serving order was also randomized. Each panelist received only two non-dairy ice cream samples at −5 °C for evaluation. A 5-point hedonic scale was employed for the evaluation of appearance, texture (structure and consistency), color, smell, and taste of non-dairy ice cream samples. After receiving the evaluation results of individual panels, the average values of individual evaluations were calculated.

### 2.9. Statiscital Analysis

Two-way analysis of variance (ANOVA) was used for statistical analysis. The effect of added antioxidant and storage time on changes in selected parameters was considered. The experiment was realized in two series. Calculations were made using Microsoft Office Excel 2007 (Microsoft Corporation, Redmond, WA, USA) and Statistica 10 (StatSoft, Warsaw, Poland) software. The post hoc Tukey’s procedure was used to find patterns and relationships between subgroups. Differences among groups were determined as statistically significant at a level of *p* ≤ 0.05. 

## 3. Results

The suggested portion of the product (90 g) can be a light dessert as it provides about 109 kcal, including about 5 g of fat, about 1 g of protein, and 16 g of carbohydrates (Table 2). Compared to other ice cream, frozen desserts, and fruit sorbets, these are typical values. Non-dairy ice cream dessert is quite a rich source of vitamin C, pyridoxine, and folic acid, and, among the minerals, they contain a lot of magnesium and potassium. It should be emphasized that the non-dairy ice cream dessert contains a negligible amount of saturated fatty acids, which is beneficial. Fat is the carrier of flavor and shapes the creamy/greasy structure of non-dairy ice cream, which is why it is readily used by non-dairy ice cream makers. In this experiment, only avocados were used in this role, and no other fatty substances were added altogether.

All analyzed desserts had a similar (~5.2 units) active acidity, stable during freezing (Table 3). The relatively low pH value is probably due to the presence of probiotics (Lb1 and Lb2 samples) or native lactic acid bacteria (CON sample) in the product, which produce acid metabolites as a result of digesting sugars. Significantly higher (*p* ≤ 0.05) oxidation-reduction potential was found in sample Lb1, but only in the study conducted before freezing the dessert. As the 8-week freezing time elapsed, differences in redox potential between all samples were not found (*p* > 0.05).

Immediately after production, the same number of LAB 7.3–8.1 log CFU/g was found in both samples with the addition of probiotic (Lb1 and Lb2) (Table 4). In the control sample, the number of LABs was significantly lower (3.9 log CFU/g). As the 8-week freezing time elapsed, differences between all trials were found at a constant significance level of *p* ≤ 0.001. After freezing, the number of LABs significantly decreased in all variants, compared to the pre-freeze test, but then remained stable, on average: 7.3 for the Lb1 sample, 5.8 for the Lb2 sample, and 1.4 for the control sample. The Lb1 variant of non-dairy ice cream dessert, in which probiotic was dissolved in 1 mL of sterile phosphate buffered saline (PBS), turned out to be the best. In this sample, the number of lactic acid bacteria was the highest, even during freezing.

In the visual evaluation of the desserts, it was noted that they had a similar tint (Table 5). The instrumental analysis of color showed some subtle differences. There were no differences in color in the results obtained after 4 weeks of freezing compared to the following weeks. The control sample was slightly lighter, but only before freezing. No difference in lightness was found after a 4-week freezing. There were found statistically significant changes in the color parameters a* (green-red chromaticity) and b* (blue-yellow chromaticity) after freezing. After 4 weeks, the color of all samples was significantly redder (*p* ≤ 0.05) and more yellow (*p* ≤ 0.05). The total color difference (∆E) of the desserts after freezing was calculated based on the individual parameter results. Color of the CON sample was the most stable (∆E = 1.7), and the probiotic samples showed clear (∆E = 3.6 or 4.0) color deviation.

According to the respondents’ assessments, the differences between the two non-dairy ice cream samples were imperceptible (Table 6). It is true that the probiotic non-dairy ice cream was rated slightly lower (by 0.1 units) than the control sample in the “appearance” and “color” parameter, but the sample was tastier (also by 0.1 units) than the control sample. The evaluators liked the structure and consistency, obtaining a high score, identical for both variants.

## 4. Discussion

The production of probiotic food with the use of fruit and vegetable raw materials as carriers, particularly fruit juices, may be associated with difficulties in the survival of bacteria. There are substances in plant raw materials that act as a carrier for microorganisms, such as fiber, vitamins, and minerals, as well as lipids [13]. The literature review shows that frozen non-dairy desserts can be an appropriate food matrix for the incorporation of lactic acid bacteria, and the probiotic effect depends on the used raw material and the individual characteristics of a strain [14]. It was found that the use PBS (Lb1), while reviving the bacteria, had a positive effect on the increase in the number of LAB. Immediately after production, no differences were observed between the variants of probiotic dessert, but, after 4 weeks, the discrepancy was almost 2 logarithmic cycles. Importantly, the use of the purelyophilisate was insufficient for the survival of bacteria under the proposed conditions. Non-dairy ice cream dessert Lb1 was the only one, among all the tested samples, which maintained the therapeutic minimum (6 log CFU of live lactic bacteria cells per 1 g of the product) defined for food enriched with probiotic bacteria, during the entire storage period [22,23]. This variant achieved high results of >8 log CFU/g immediately after production and >7 log CFU/g after the end of frozen storage. It should be noticed that, if the product were consumed, the bacteria would be additionally exposed to the harsh conditions of the gastrointestinal tract and could reduce survival by several log scales before reaching their destination. In the studies by Szydłowska and Kołożyn-Krajewska [24], a significant effect of storage time was also observed on the reduction of the survival rate of *Lactobacillus rhamnosus* LOCK900 in fruit and tea sorbets. The product, after 12 weeks of storage, was characterized by the content of live bacterial cells at the level of >7 log CFU/cm^3^, similar to obtained in this experiment. According to Mohammadi et al. [12], the final survival rate is influenced by many complex factors, including the type of sugar and its concentration, temperature and freezing speed, the type of probiotic used, and the total storage time. 

TVC is an important determinant of microbial quality, and results are confirmed by the results for the number of lactic acid bacteria. It was shown that, in the sample with the probiotic activated in PBS solution, the total number of bacteria remained unchanged throughout the storage period. The values of both, the sample with the pure lyophilizate of bacteria and the control sample free from probiotic LAB, decreased over time. In the studies of Senanayake et al. [25] also obtained an excess amount of TVC in the sample of fruit ice cream enriched with *Lactobacillus acidophilus* compared to the reference sample.

From the observed results of the acidity, it can be clearly noticed that, immediately after production, the probiotic sample with PBS (Lb1) reached the lowest pH, which proves the high acidity and undeniable fermentation activity of the lactic acid bacteria. Although the value increased after the first 4 weeks of freezing, it remained at the same level after the next 4 weeks, which may suggest the inhibitory effect of low temperature. Szydłowska and Kołożyn-Krajewska [24] noticed no significant influence of cooling conditions on the change in the value of sorbets with the addition of *Lactobacillus rhamnosus* LOCK900. Moreover, Nousia et al. [26], after 45-week storage of milk ice cream enriched with *Lactobacillus acidophilus* LMGP-21381, also did not notice significant differences from the original values. Ervina et al. found that the addition of avocado to non-dairy ice cream reduces the acidity of the product, but no statistically significant differences were found compared to the control sample [15]. This is related to the susceptibility of probiotic bacteria to dying under conditions of too low pH. Therefore, there is a need to select suitably resistant species [12]. However, it is possible to effectively improve the growth potential of bacteria by using raw materials rich in prebiotics, as proven by Iraporda et al. [27], who analyzed the effect of inulin isolated from Jerusalem artichoke on the survival and functional properties of various *Lactobacillus* strains. There was checked the survival of bacteria under simulated gastrointestinal digestion. Inulin had a positive effect on the growth of bacteria survival and improved their resistance to gastrointestinal conditions during simulated digestion. 

The oxidation-reduction potential is an important indicator for monitoring and estimating oxidative changes in the product. In our study, this parameter is of particular importance due to the used raw material—avocado, which mainly contains monounsaturated fatty acids susceptible to oxidation processes [15]. The fact that no significant changes were observed between the values obtained in the 4th and 8th week of freezing storage may indicate the production of metabolites by the used LAB strain that affect the stability of reducing properties during process [28].

LAB can find application in the production of fermented plant food if they meet the important aspect of maintaining the desired sensory quality [24]. The panelists who performed the organoleptic evaluation of desserts did not recognize any of the products as particularly outstanding. In the studies of Homayouni et al. [14], no statistically significant differences were found in terms of the organoleptic evaluation between the *Lactobacillus casei* fermented plant-based ice cream (soybean) and the unfermented control product. On the other hand, in the studies by Norouzi et al. [29], fermentation of soy ice cream with *Lactobacillus paracasei* had a positive effect on the organoleptic characteristics of desserts. The authors’ own research positively accepted the fact that the taste and smell of probiotic desserts were highly rated. It is also promising that the acidic reaction of ice cream with probiotics was not perceptible by the respondents. It is assumed that the plant material is capable of masking undesirable odors that may appear during fermentation processes [13]. The results of the organoleptic evaluation in terms of color (Table 6) were reflected in the values obtained during a calculation of the color deviations (Table 5) between the samples.

Ice cream enriched with probiotics, as a product intended for people who respect the impact of nutrition on the body, should have an attractive nutritional value. It should be noted that conventional ice cream contains mainly milk fat rich in saturated fatty acids. Avocados, which were a component of the ice cream in this study, are distinguished by a high content of monounsaturated fatty acids; moreover, they contain many antioxidant compounds that can be used in an anti-inflammatory diet [15]. The energy value of traditional ice cream is higher than that of sorbets, and it is usually 100–200 kcal/100 g in the mass of ice cream, without a wafer. The calories are increased by the wafer, added sugars, and additives. Kalicka et al. [23] made an attempt to produce milk ice cream enriched with *Bifidobacterium lactis* BB-12 sweetened with polyols, not with sucrose, for the creation of sugar-free product. Ice cream produced in this experiment does not contain any additional amount of sugar other than naturally present in the fruit. The main source of carbohydrates in products are bananas, which have a high glycemic index (GI), but the presence of avocado fat may slow down the absorption of glucose into the blood and be a factor lowering the predicted GI of a meal [30]. Non-dairy ice cream desserts with probiotic LAB created in our own research, as products manufactured without use of animal ingredients, respond to the needs of vegans and vegetarians.

## 5. Conclusions

It was found that the use of the probiotic did not deteriorate the overall quality of the product, including sensory evaluation. The non-dairy ice cream dessert based on avocado with the addition of probiotic LAB was similar, both terms of physicochemical properties and organoleptic evaluation, to the control sample. Both mean pH values (5.2 ± 0.1) and mean oxidation-reduction potential values (454 ± 11 mV) were similar in all variants. The *L. plantarum* 299v probiotic strain is presumably able to multiply and survive in non-dairy ice cream dessert based on avocado. Dissolving the bacterial lyophilizate in PBS improved the viability of the bacteria in the product. After several weeks of freezing, the number of LABs in this variant was, on average, 7.4 log CFU/g and was over 1 logarithmic order higher than in the variant without PBS addition. It is true that the presence of exactly *L. plantarum* 299v in the dessert was not determined. However, the analysis of lactic acid bacteria, to which this strain belongs, was performed. As differences in the number of LABs between the dessert with the probiotic and the control sample were noticed, there is a strong supposition that the *L. plantarum* is in the product. Further studies are needed to confirm this hypothesis. The presence of probiotic bacteria in the non-dairy ice cream dessert and their survival in the freezing process is the most important factor and the starting point for further research work on composing a potentially functional fruit dessert enriched with probiotics.

## Figures and Tables

**Table 1:** Comparison of the nutritional value of avocado and banana based on the data in the food nutritional table [16].

Nutritional Value per 100 g	Avocado	Banana
Total fat (g) - saturated fat (g) - monounsaturated fat (g) - polyunsaturated fat (g)	15.3 1.80 11.5 1.33	0.30 0.12 0.03 0.06
Total Carbohydrates (g) - dietary fiber (g) - total sugars (g) - fructose (g) - saccharose (g)	7.40 3.30 4.10 0.20 0.10	23.5 1.70 21.8 3.70 11.1
Protein (g) - exogenous amino acids (g)	2.00 0.84	1.00 0.50
Vitamins - folates (μg) - niacin (mg) - pyridoxine (mg) - vitamin A (μg) - vitamin C (mg) - vitamin E (mg)	62.0 1.90 0.26 7.00 8.00 1.30	22.0 0.50 0.36 8.00 9.00 0.27
Minerals - calcium (mg) - iodine (μg) - magnesium (mg) - phosphorus (mg) - potassium (mg) - sodium (mg)	11.0 2.00 39.0 41.0 600 10.0	6.00 2.80 33.0 20.0 395 1.00

**Table 2:** Nutrition facts of a non-dairy ice cream dessert in one (90 g) serving.

Nutrition Facts in Serving Size (90 g)	Non-Dairy Ice Cream Dessert	% Daily Value ^1^
Energy value - kcal - kJ	109 456	5 5
Total fat (g) - saturated fat (g)	4.77 0.61	6 3
Total Carbohydrates (g) - dietary fiber (g) - sugars (g)	16.3 2.01 14.3	6 7 16
Protein (g)	1.20	2
Vitamins - folates (ug) - niacin (mg) - pyridoxine (mg) - vitamin A (ug) - vitamin C (mg) - vitamin E (mg)	31.8 0.87 0.29 6.90 7.80 0.55	8 5 17 1 9 4
Minerals - calcium (mg) - iodine (ug) - magnesium (mg) - phosphorus (mg) - potassium (mg) - sodium (mg)	6.90 2.28 31.5 24.3 417 3.6	1 2 8 2 9 0

^1^ The % daily value tells how much a nutrient in a serving of food contributes to a daily diet, and 2000 calories a day is used for general nutrition advice. For macronutrients data from EU, Regulation 1169/2011 [20] were used. For vitamins and minerals, the FDA standards were used [21].

**Table 3:** Physicochemical analysis of the non-dairy ice cream dessert during freezing.

Test	Time	Samples		
		Lb1	Lb2	CON
pH value (−)	before freezing	5.1 ± 0.0 ^bA^	5.2 ± 0.0 ^aA^	5.2 ± 0.0 ^aA^
	after 4 weeks of freezing	5.2 ± 0.1 ^aB^	5.3 ± 0.0 ^aB^	5.3 ± 0.0 ^aB^
	after 8 weeks of freezing	5.1 ± 0.0 ^aB^	5.2 ± 0.0 ^bB^	5.2 ± 0.1 ^bB^
Oxidation-reduction potential (mV)	before freezing	473 ± 2.4 ^aA^	466 ± 2.5 ^bA^	466 ± 1.3 ^bA^
	after 4 weeks of freezing	446 ± 2.9 ^aB^	441 ± 4.3 ^aB^	447 ± 0.8 ^aB^
	after 8 weeks of freezing	451 ± 4.0 ^aB^	445 ± 1.4 ^aB^	451 ± 3.9 ^aB^

Mean values marked with the same lowercase letter (Lb1, Lb2, and CON) and the same uppercase letter (freezing time) do not differ significantly at *p* ≤ 0.05.

**Table 4:** Microbial analysis of the non-dairy ice cream dessert during freezing.

Test	Time	Samples		
		Lb1	Lb2	CON
LAB (log CFU/g)	before freezing	8.1 ± 0.3 ^aA^	7.3 ± 0.2 ^aA^	3.9 ± 1.2 ^bA^
	after 2 weeks of freezing	7.2 ± 0.3 ^aB^	5.7 ± 0.3 ^bB^	1.0 ± 0.2 ^cB^
	after 4 weeks of freezing	7.5 ± 0.2 ^aB^	5.9 ± 0.3 ^bB^	1.8 ± 0.2 ^cB^
	after 8 weeks of freezing	7.5 ± 0.3 ^aB^	5.9 ± 0.3 ^bB^	1.3 ± 0.2 ^cB^
TVC (log CFU/g)	before freezing	8.0 ± 0.3 ^aA^	7.0 ± 0.3 ^bA^	4.7 ± 0.2 ^cA^
	after 2 weeks of freezing	7.3 ± 0.3 ^aA^	5.8 ± 0.3 ^bB^	3.1 ± 0.2 ^cB^
	after 4 weeks of freezing	7.3 ± 0.3 ^aA^	5.8 ± 0.3 ^bB^	3.3 ± 0.3 ^cB^
	after 8 weeks of freezing	7.3 ± 0.3 ^aA^	5.8 ± 0.3 ^bB^	2.9 ± 0.3 ^cB^

Mean values marked with the same lowercase letter (Lb1, Lb2, and CON) and the same uppercase letter (freezing time) do not differ significantly at *p* ≤ 0.05.

**Table 5:** Color parameters of the non-dairy ice cream dessert during freezing.

Test	Time	Samples		
		Lb1	Lb2	CON
L* parameter (−)	before freezing	58.6 ± 3.5 ^aA^	59.0 ± 2.4 ^aA^	61.4 ± 1.1 ^bA^
	after 4 weeks of freezing	59.0 ± 5.2 ^aA^	61.0 ± 2.1 ^aB^	60.3 ± 2.0 ^aA^
a* parameter (−)	before freezing	2.8 ± 0.2 ^aA^	3.1 ± 0.2 ^bA^	3.1 ± 0.2 ^bA^
	after 4 weeks of freezing	4.3 ± 0.2 ^aB^	4.0 ± 0.5 ^aB^	4.1 ± 0.7 ^aB^
b* parameter (−)	before freezing	23.6 ± 3.7 ^aA^	25.8 ± 1.7 ^aA^	28.7 ± 1.6 ^bA^
	after 4 weeks of freezing	27.6 ± 2.0 ^aB^	28.7 ± 1.6 ^abB^	29.5 ± 2.3 ^bA^
Δ*E* (−)		4.0	3.6	1.7

Mean values marked with the same lowercase letter (Lb1, Lb2, and CON) and the same uppercase letter (freezing time) do not differ significantly at *p* ≤ 0.05. The results after 8 weeks of freezing are not included in the table because they did not differ from those after 4 weeks of freezing.

**Table 6:** Sensory evaluation of the non-dairy ice cream dessert (n = 31).

Characteristic	Lb1	CON
Appearance	3.12	3.25
Color	3.96	4.03
Structure and consistency	4.35	4.35
Smell and taste	4.54	4.45
Overall quality	4.0 ± 0.6	4.0 ± 0.5

## Data Availability

Not applicable.
